# Integrated multi-omic analysis and experiment reveals the role of endoplasmic reticulum stress in lung adenocarcinoma

**DOI:** 10.1186/s12920-023-01785-4

**Published:** 2024-01-02

**Authors:** Ying Liu, Wei Lin, Hongyan Qian, Ying Yang, Xuan Zhou, Chen Wu, Xiaoxia Pan, Yuan Liu, Gaoren Wang

**Affiliations:** 1 Cancer Research Center Nantong, Affiliated Tumor Hospital of Nantong University, Medical School of Nantong University, Nantong, China; 2 Jiangsu Key Laboratory of Neuropsychiatric Diseases and Institute of Neuroscience, Soochow University, Suzhou, China

**Keywords:** Lung adenocarcinoma, Endoplasmic reticulum, Prognosis, Therapy, Immune infiltration

## Abstract

**Background:**

Lung cancer is a highly prevalent malignancy worldwide and is associated with high mortality rates. While the involvement of endoplasmic reticulum (ER) stress in the development of lung adenocarcinoma (LUAD) has been established, the underlying mechanism remains unclear.

**Methods:**

In this study, we utilized data from The Cancer Genome Atlas (TCGA) to identify differentially expressed endoplasmic reticulum stress-related genes (ERSRGs) between LUAD and normal tissues. We performed various bioinformatics analyses to investigate the biological functions of these ERSRGs. Using LASSO analysis and multivariate stepwise regression, we constructed a novel prognostic model based on the ERSRGs. We further validated the performance of the model using two independent datasets from the Gene Expression Omnibus (GEO). Additionally, we conducted functional enrichment analysis, immune checkpoint analysis, and immune infiltration analysis and drug sensitivity analysis of LUAD patients to explore the potential biological function of the model. Furthermore, we conducted a battery of experiments to verify the expression of ERSRGs in a real-world cohort.

**Results:**

We identified 106 ERSRGs associated with LUAD, which allowed us to classify LUAD patients into two subtypes based on gene expression differences. Using six prognostic genes (NUPR1, RHBDD2, VCP, BAK1, EIF2AK3, MBTPS2), we constructed a prognostic model that exhibited excellent predictive performance in the training dataset and was successfully validated in two independent external datasets. The risk score derived from this model emerged as an independent prognostic factor for LUAD. Confirmation of the linkage between this risk model and immune infiltration was affirmed through the utilization of Gene Set Enrichment Analysis (GSEA), Gene Ontology (GO), and Kyoto Encyclopedia of Genes and Genomes (KEGG) enrichment analyses. The q-PCR results verified significant differences in the expression of prognostic genes between cancer and paracancer tissues. Notably, the protein expression of NUPR1, as determined by immunohistochemistry (IHC), exhibited an opposite pattern compared to the mRNA expression patterns.

**Conclusion:**

This study establishes a novel prognostic model for LUAD based on six ER stress-related genes, facilitating the prediction of LUAD prognosis. Additionally, NUPR1 was identified as a potential regulator of stress in LUAD.

**Supplementary Information:**

The online version contains supplementary material available at 10.1186/s12920-023-01785-4.

## Introduction

Lung cancer poses a significant worldwide health issue, wherein non-small cell lung cancer (NSCLC) comprises approximately 80-85% of the reported instances [1]. According to global cancer statistics, over 2 million new cases of lung cancer are diagnosed each year [2, 3]. Among the various subtypes of NSCLC, lung adenocarcinoma (LUAD) represented approximately 55-60% of cases [4]. Despite significant advancements in immune checkpoint inhibitors and anti-angiogenesis therapies that have improved survival rates, the 5-year survival rate for patients remains around 20% [5–7]. While several conventional clinical models have been utilized to predict the prognosis of LUAD, the inherent heterogeneity of the disease limits their ability to provide accurate results [8]. Consequently, there is a need to develop new prognostic signatures that can enhance the prognosis assessment for LUAD patients.

The endoplasmic reticulum (ER) is a multifunctional organelle responsible for protein folding, lipid biosynthesis, and calcium storage [9, 10]. Notably, it serves as a central hub for protein quality control, enabling adaptation to adverse synthesis, external stimuli, and other detrimental events. ER stress has been implicated in the development and progression of various human malignancies, as it affects multiple cancer hallmarks [11]. External adverse factors can disrupt the integrity of the ER, leading to the accumulation of unfolded or misfolded proteins within its lumen, a condition known as ER stress. This triggers the activation of the unfolded protein response (UPR) [12, 13]. In several cancer types, overexpression of ER stress indicators has been associated with poor prognosis and clinical outcomes [14]. Wei et al. conducted a study confirming that the activation of ER stress signals plays a significant role in the initiation and progression of liver cancer [15]. Furthermore, they discovered that suppressing the ER stress response enhances cellular susceptibility to cisplatin therapy in NSCLC [16]. A recent study demonstrated that ER stress induces oral squamous cell cancer cells to secrete exosome PD-L1, leading to upregulated PD-L1 expression in macrophages and driving the polarization of M2 macrophages [17]. However, a comprehensive understanding of ER stress in LUAD, including the interplay between ER stress regulators and the tumor immune microenvironment (TIME), remains elusive.

To investigate and assess the clinical significance of ER stress in LUAD, a comprehensive analysis of endoplasmic reticulum stress-related genes (ERSRGs) was conducted in this study. Additionally, a predictive model based on ERSRGs was constructed to evaluate its prognostic value in LUAD patients. Functional enrichment analysis revealed a correlation between ERSRGs and immune infiltration. The findings of this study offer insights into the potential molecular mechanisms underlying LUAD and provide valuable prognostic information for clinical management.

## Materials and methods

### Data collection

The clinical information and RNA sequencing data for the bioinformatics analysis were obtained from publicly available databases, namely The Cancer Genome Atlas (TCGA, https://portal.gdc.cancer.gov/). A total of 453 samples diagnosed with LUAD were included in the training set, ensuring the availability of complete clinical information, including survival time, survival status, age, and gender. Additionally, to further validate the findings, datasets consisting of 352 patients diagnosed with LUAD was acquired from the Gene Expression Omnibus (GEO) database (https://www.ncbi.nlm.nih.gov/geo/). These datasets comprised of two independent cohorts, namely GSE31210 (246 samples) and GSE37745 (106 samples), from which mRNA expression matrices were extracted. Subsequently, these datasets were integrated and defined as the validation sets.

### Clinical sample collection

For quantitative polymerase chain reaction (Q-PCR) experiments, tissue samples were obtained from a cohort of eight patients who underwent pulmonary lobectomy at the Affiliated Cancer Hospital of Nantong University between August 2022 and April 2023. The tissue samples comprised both LUAD and paired collateral cancer specimens. Additionally, six patients diagnosed with LUAD were included for immunohistochemistry experiments, and their samples were sourced from Nantong Tumor Hospital. Prior to their participation in the study, all patients provided written informed consent. The research protocol was approved by the Ethics Committee of the Affiliated Cancer Hospital of Nantong University.

### Quantitative polymerase chain reaction

Eight pairs of cancer and adjacent non-cancerous tissues were collected for qPCR. Total RNA extraction was conducted utilizing TRIzol reagent (Thermo Fisher SCIENTIFIC, USA), following the manufacturer’s instructions. Reverse transcription of mRNA was accomplished using the EvoM-MLV reverse transcription kit (Accurate Biology, China) [18]. For reverse transcription of mRNA, the HiScript III RT SuperMix for qPCR (Vazyme, China) was employed. The primers used in this study were procured from Sangon Biotech, and their specific sequences are provided in Table 1.

**Table 1:** The primer sequence of 6 genes

GENE	PRIMER	SEQUENCE (5’-3’)
BAK1	Forward	atc aac cga cgc tat gac tca gag
	Reverse	aca ggc tgg tgg caa tct tgg
NUPR1	Forward	agg aac aga tgc acg tca gac tac
	Reverse	gat tag gct gga ctc aag gga agg
VCP	Forward	tcc tgt tgc ctc acc ctt tgt c
	Reverse	gcc tag cct tac cgt cca. cat c
RHBDD2	Forward	atc ttc gcc atc ttc tcc gct atc
	Reverse	cga gaa cgg acg gtg gtg ac
MBTPS2	Forward	cgg gtc tcc tga cag atc aca ag
	Reverse	aga tgt cct gag cag cac aag ag
EIF2AK3	Forward	tgg atg atg tgg tca agg ttg gag
	Reverse	gtg tct ggc ata agc tgg cat tg

### Western blot

Besa-2b, A549, H1299, H1975, and PC9 cell cultures (MeisenCTCC, China) were collected and underwent lysis using phenylmethylsulfonyl fluoride (1:100, Beyotime, Shanghai, China) in conjunction with cell lysis buffer. The protein was then separated using electrophoresis and transferred to a polyvinylidene fluoride (PVDF) membrane (Invitrogen, USA). Following a 2-hour blocking step with 5% skim milk, the PVDF membranes underwent an incubation period at 4 °C overnight with NUPR1 antibody (1:400, Proteintech, China). After three wash cycles, blots were incubated with horseradish peroxidase-conjugated secondary antibodies (1:1000, Proteintech, China). The determination of relative expression involved dividing the target protein band density by the density of Tubulin.

### Immunohistochemistry

The tumor tissue microarray, obtained from the Affiliated Tumor Hospital of Nantong University, was utilized for the validation of the queue. Immunohistochemistry (IHC) was performed following previously established protocols [19]. In brief, the tissue sections were incubated with a primary Anti-NUPR1 antibody (dilution 1:100; catalog number 15056-1-AP, Proteintech, China) and subsequently processed using the appropriate detection system. A scanning microscope (Nikon, Japan) was employed for capturing high-resolution images of the stained sections. The evaluation of NUPR1 staining was conducted by two independent pathologists, who were blinded to the corresponding clinical information. They assessed the staining intensity, distribution, and cellular localization of NUPR1 in a semi-quantitative manner using established scoring criteria. Any discrepancies between the two pathologists were resolved through consensus discussion.

### Differentially expressed genes associated with ER stress

Differential gene expression analysis was performed on the TCGA-LUAD dataset using the R-package “limma” to identify genes that were differentially expressed between LUAD and healthy samples. The criteria for differential expression were set as |log_2_FoldChange| > 1 and adj. *p* < 0.05. The resulting gene set was then intersected with the ERSRGs, leading to the identification of 106 ER stress-related differentially expressed genes (DEGs).

To further identify ER stress-related prognostic genes, univariate Cox regression analysis was conducted on the TCGA-LUAD dataset. The genes were subjected to least absolute shrinkage and selection operator (LASSO) regression using the R package “glmnet” to identify genes associated with overall survival (OS). Multiple factor stepwise regression was then applied, and the smallest lambda value was considered as the optimal value. Risk score: ${\sum }_{\text{i}=1}^{\text{n}}=$ Coef (gene) * Expression (gene). A nomogram was constructed using the R packages “rms” and “survival” to predict the survival of LUAD patients. The nomogram included various variables such as age, sex, TN staging, histological grading, and risk score. The accuracy of the nomogram was verified by plotting calibration curves at 1-year, 3-year, and 5-year intervals using the R package “rms”.

Based on the median risk score, patients were divided into high-risk and low-risk subgroups. Kaplan-Meier (K-M) curves, time-dependent receiver operating characteristic (ROC), and a riskscore plot were generated through multivariate Cox regression analysis to illustrate the distribution and survival status of LUAD patients in the two risk groups.

### Consensus clustering analysis

Cluster analysis was performed on a cohort of 106 patients using the Pearson correlation distance measure. To ensure robustness, the clustering process was repeated 10 times on 80% of the samples. The optimal number of clusters was determined by analyzing the empirical cumulative distribution function graph.

### Functional enrichment analysis

Gene Ontology (GO) analysis, Kyoto Encyclopedia of Genes and Genomes (KEGG) analysis, and Gene Set Enrichment Analysis (GSEA) were employed to investigate potential mechanisms and pathways associated with the two clusters and riskscore subgroups [20].

### Immune infiltration analysis and single cell sequencing analysis

The degree of immune cell infiltration and the presence of immune checkpoints were compared across different riskscore subgroups. The “ESTIMATE” package was utilized to calculate the stromal score, immune score, and tumor purity for LUAD patients. To ensure the stability of the results, the “TIMER”, “EPIC”, and “MCP-counter” tools were employed.

Additionally, single-cell RNA sequencing data from the GSE127465 dataset was screened to identify relevant information. The TISCH platform, specifically its built-in tSNE algorithm available at http://tisch1.comp-genomics.org/, was employed for dimensionality reduction and visualization of the identified clusters.

### Cell culture

Beas-2b cells, representing a human normal lung epithelial cell line, underwent cultivation in RPMI 1640 medium supplemented with 10% fetal calf serum (Gibco, Grand Island, NY, USA) and 1% Penicillin-Streptomycin (NCM Biotech, China). H1299, H1975, and PC9 cells were cultured in RPMI 1640 medium with 10% fetal bovine serum (FBS) at 37 °C. Similarly, A549 cells were nurtured in F-12 K medium (Gibco, Grand Island, NY, USA) supplemented with 10% FBS. Cell lines were meticulously maintained in their respective culture media to facilitate optimal growth and experimental conditions.

### Cell counting Kit-8 assay and transwell

H1299 cells were inoculated in 96-well plates at a density of 5*10^3^ cells per well. Subsequently, the cells were exposed to Trifluoperazine dihydrochloride (MedChemExpress, China) at a concentration of 20 μmol /mL and incubated at 37 °C for 24, 48, and 72 h. Following the respective incubation periods, an absorbance reading at 450 nm was obtained using a microplate reader (Thermo Fisher Scientific, Waltham, MA, USA). This measurement was conducted after a 2-hour incubation at 37 °C with 10 μL of Cell Counting Kit-8 (CCK-8; Bimake, Houston, TX, USA) reagent in each well. To standardize the concentration of H1299 cells to 1 × 10^5^/mL, serum-free RPMI-1640 medium was employed. The upper chamber received 200 μL of RPMI-1640 medium containing 10% fetal bovine serum, while the lower chamber was supplemented with 600 μL of RPMI-1640 medium containing 10% fetal bovine serum. The cell culture was maintained at 37℃ with 5% CO_2_ for 24 h. After chamber removal, the cells in the upper chamber were delicately swabbed with a cotton applicator, fixed with 4% paraformaldehyde for 15 min, stained with crystal violet at room temperature for 25 min, and excess staining solution was rinsed off with PBS. Observation of cells that traversed the membrane was performed under a microscope. For each sample, three randomly selected fields of view were photographed and enumerated, and the average value was computed.

### Drug sensitivity analysis

The half-maximum inhibitory concentration (IC50) metrics for chemotherapeutic agents were acquired from the Genomics of Cancer Drug Sensitivity (GDSC) database, accessible at https://www.cancerrxgene.org/. Subsequently, “PRrophytic” R package was performed to calculate the drug susceptibility between different subgroups in R software. The outcomes are visually represented through box plots.

### Statistical analysis

Statistical analyses and data visualization were conducted using R software version 4.1.0 and GraphPad Prism version 9.5.1. Both univariate and multivariate Cox regression analyses were employed to examine the impact of various factors on the prognosis of LUAD. Statistical significance was defined as *p*-values < 0.05.

## Results

### The screening and characterization of ERSRGs in LUAD

**Fig. 1:**
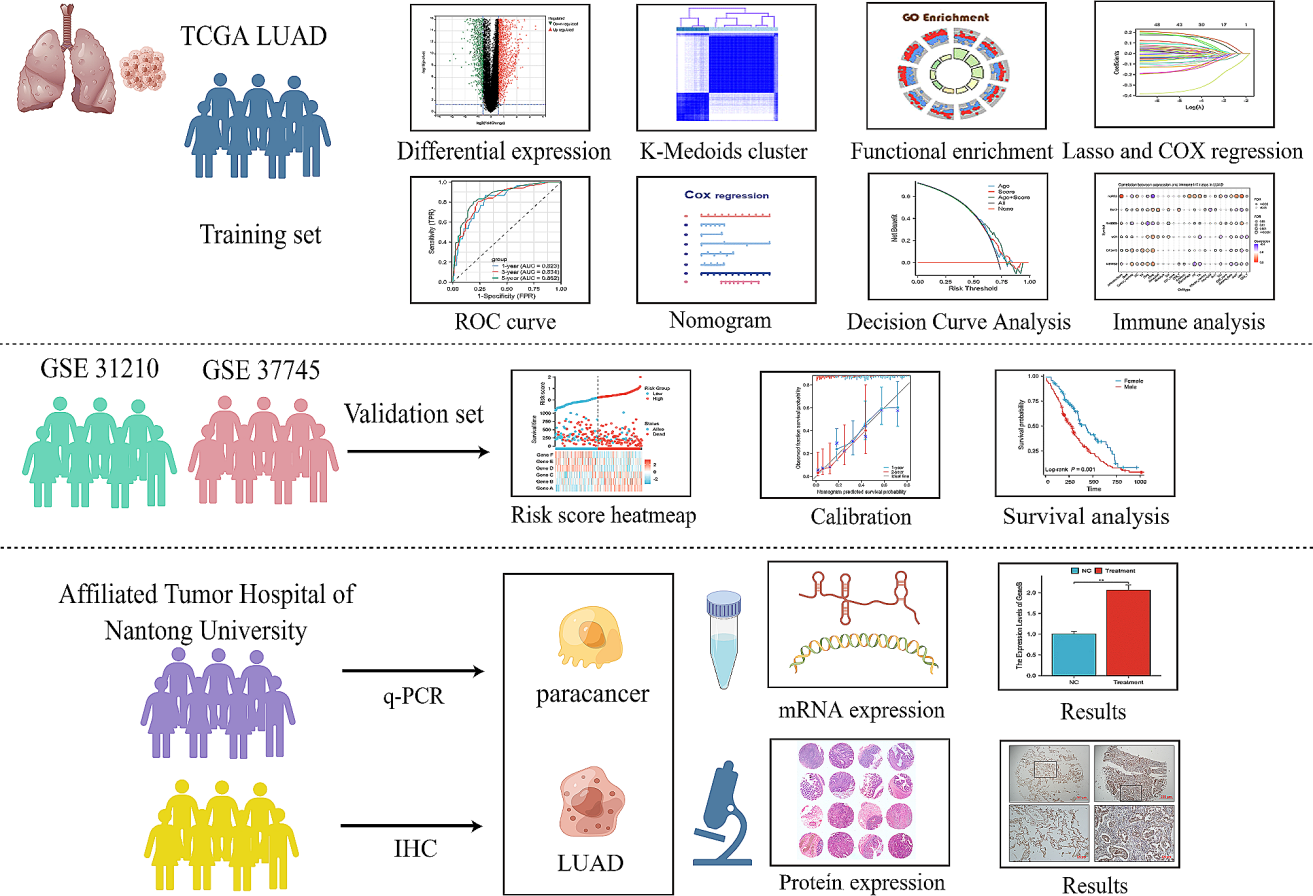
Flow Chart of this Research

**Fig. 2:**
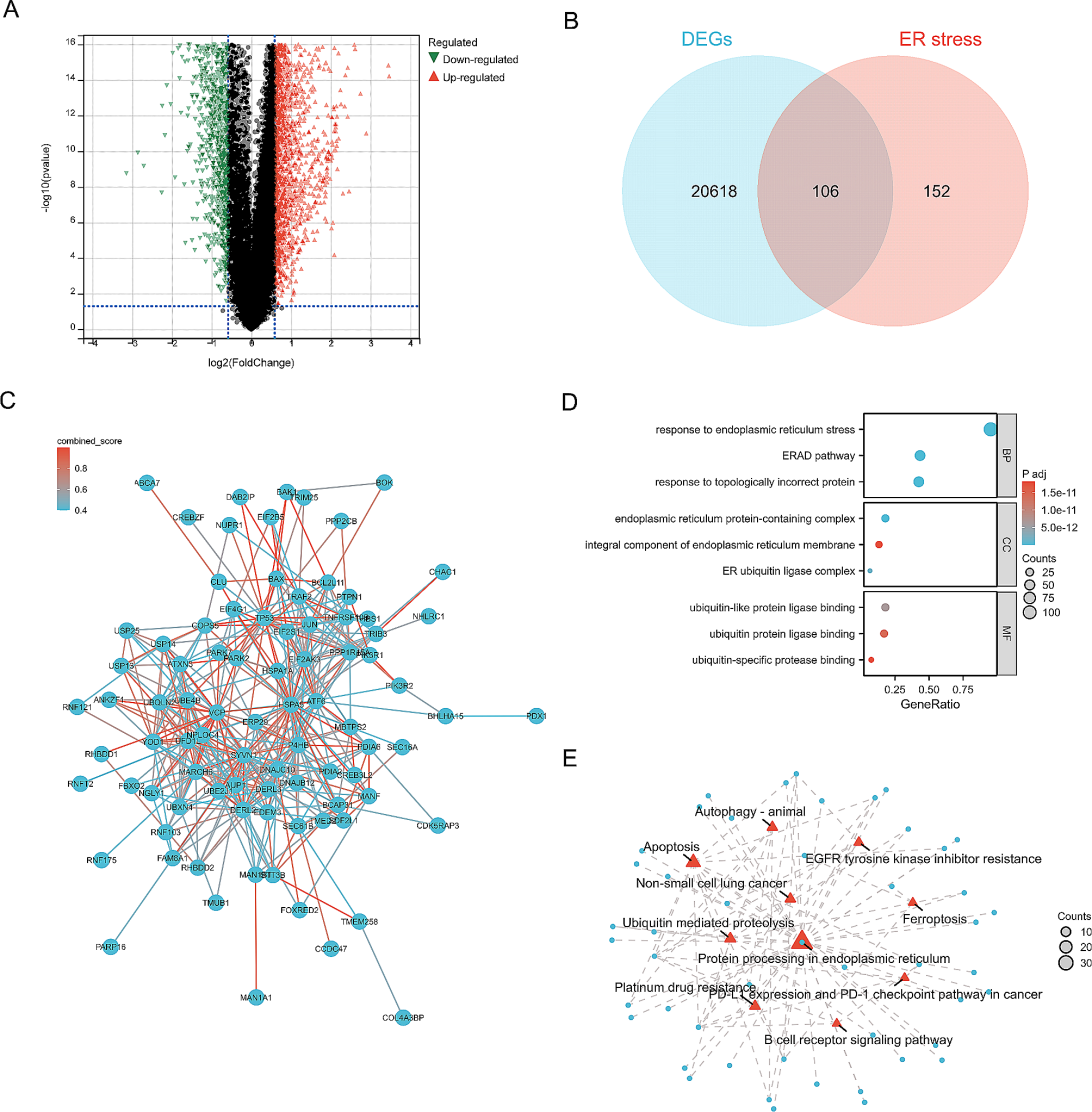
The screening and characterization of ERSRGs in LUAD. (**A**) Volcano plot showing DEGs between LUAD and control samples. (**B**) Venn diagram showing the intersection of DEGs and ER stress-related genes. (**C**) The PPI network shows the interactions of the ERSRGs in LUAD. (**D**) GO functional enrichment analysis of the intersecting genes with the top three of BP, CC and MF terms (**E**) The KEGG enrichment results are displayed, and the node size represents the number of genes enriched

The study flow-chart is shown in Fig. 1. Differential expression analysis between the LUAD and control groups (TCGA cohort) was conducted using the limma package in R, leading to the identification of 20,724 DEGs. Among these DEGs, 14,019 were up-regulated, while 6,705 were down-regulated (Fig. 2A). To investigate the potential involvement of ER stress in LUAD, a Venn analysis was performed to identify the overlap between the LUAD-related DEGs and ER stress genes (Fig. 2B). The interaction network among the 106 genes is visualized in Fig. 2C, and the intersecting genes were functionally annotated using GO and KEGG analyses. As depicted in Fig. 2D, these genes are enriched in various biological processes, such as response to ER stress, response to topologically incorrect protein, ER-associated Degradation (ERAD) pathway, cell components including ER protein-containing complex, integral component of ER membrane, ER ubiquitin ligase complex, and molecular functions such as ubiquitin-like protein ligase binding, ubiquitin ligase binding and ubiquitin protease binding. The critical functions of the ERSRGs include ubiquitin mediated proteolysis, protein processing in ER, B cell receptor signaling pathway and so on (Fig. 2E).

### Consensus clustering analysis of ERSRGs in LUAD

**Fig. 3:**
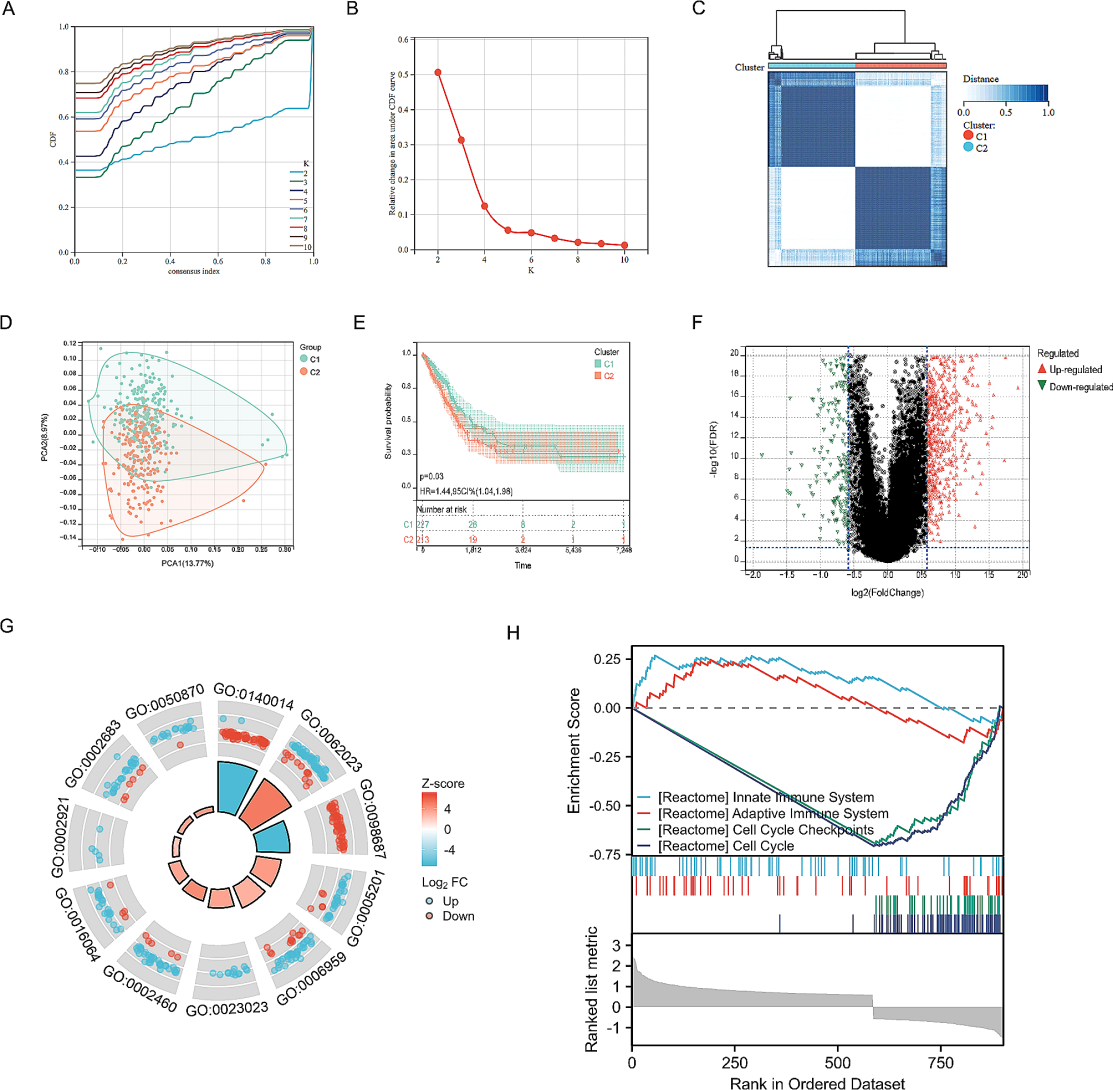
Clustering analysis of endoplasmic reticulum stress related gene in patients with LUAD. (**A**, **B**) When k = 2, the consistent clustering Delta area curve shows the best model construction. (**C**) The cluster diagram of the consistency cluster analysis of ERSRGs in 453 samples in TCGA LUAD. (**D**) PCA analysis of two clusters. (**E**) KM curve of survival between cluster 1 and cluster 2. (**F**) Volcano map of differential gene expression between two clusters. (**G**) The GO enrichments in two clusters. (**H**) GSEA analysis between cluster 1 and cluster 2

Based on the identified set of 106 genes, the cohort of LUAD patients from the training queue (TCGA cohort) was subjected to Cluster Analysis to classify them into two subgroups. Optimal stability was observed when K = 2, resulting in the classification of 232 patients into cluster 1 and 221 patients into cluster 2 (Fig. 3A-C). Principal component analysis demonstrated a distinct separation of samples into two clusters (Fig. 3D). Notably, the OS rate of patients in cluster 2 was significantly higher than that of cluster 1 (*P* = 0.03; Fig. 3E). These findings supported the subdivision of LUAD patients into two distinct molecular subtypes associated with differing survival outcomes. Furthermore, a volcano plot depicting the logFC and FDR values of 620 upregulated genes and 321 downregulated genes across the two clusters was generated (Fig. 3F). Subsequent GO enrichment analysis revealed that these genes were significantly enriched in specific molecular processes, such as mitotic sister chromatid segregation, humoral immune response, regulation of humoral immune response, condensed chromosome, centromeric region, and others (Fig. 3G; Table 2). Additionally, GSEA revealed that the enriched pathways were predominantly associated with immune infiltration (Fig. 3H). Consequently, it is reasonable to hypothesize that the influence of risk scores may impact the prognosis of LUAD by modulating the immune microenvironment.

**Table 2:** GO enrichment analysis of ERSRGs

Term	ID	Description	p.adjust
BP	GO: 0140014	mitotic nuclear division	2.78E-18
BP	GO: 0006959	humoral immune response	8.98E-09
BP	GO: 0002460	adaptive immune response	2.86E-05
BP	GO: 0016064	immunoglobulin mediated immune response	9.57E-05
BP	GO: 0002921	negative regulation of humoral immune response	0.009377062
BP	GO: 0002683	negative regulation of immune system process	0.021809421
BP	GO: 0050870	positive regulation of T cell activation	0.022529834
CC	GO: 0062023	collagen-containing extracellular matrix	4.07E-17
CC	GO: 0098687	chromosomal region	3.47-12
MF	GO: 0005201	extracellular matrix structural constituent	2.43E-09
MF	GO: 0023023	MHC protein complex binding	4.78E-07

### Prognostic signature was constructed and validation based on ERSRGs in LUAD

**Fig. 4:**
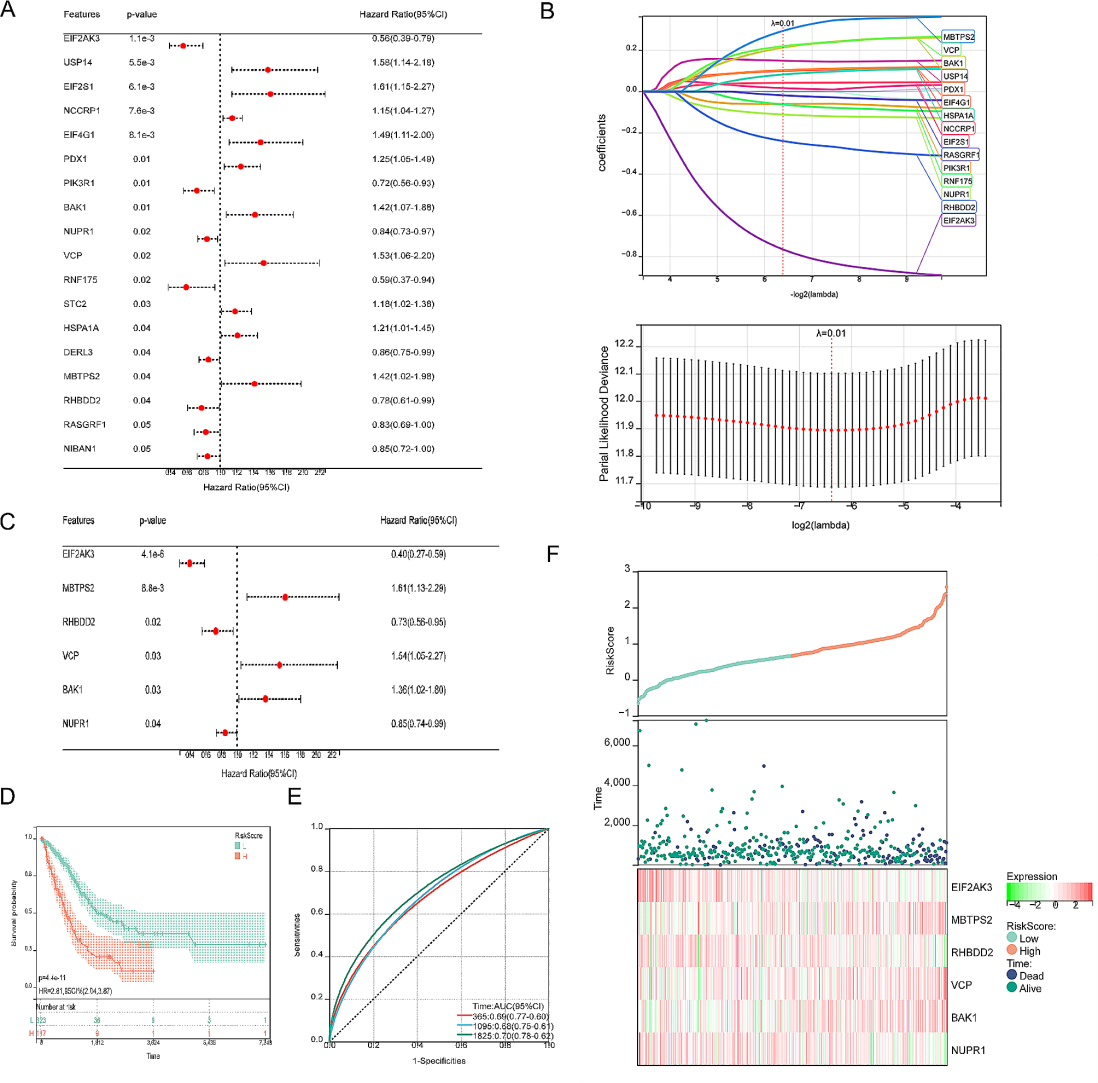
Identification of ERSRGs-signature. Univariate analysis (**A**), LASSO analysis (**B**) and stepwise Cox algorithm (**C**) were used to identified a prognostic ER stress-related signature. (**D**) Kaplan-Meier survival curves between high and low subgroups. (**E**) For this ERSRGs-signature, the area under the ROC curve is 0.69 (1 years), 0.68 (3 years), 0.70 (5 years). (**F**) Riskscore plot showed the relationship among status, survival time and ERSRGs expression

The initial step in developing a prognostic model involved the identification of candidate prognostic ERSRGs through univariate Cox regression analysis. As depicted in Fig. 4A, the OS of LUAD patients exhibited a significant correlation with 18 ERSRGs. Subsequently, LASSO analysis was employed to detect and screen 15 DEGs associated with ER stress (Fig. 4B). Furthermore, a multiple factor stepwise regression analysis was performed, resulting in the selection of 6 genes for constructing the prognostic model (Fig. 4C). The risk scoring model was established using the following formula: Riskscore = (-0.894871321) × EIF2AK3 + (0.268950582) × BAK1 + (-0.127961954) × NUPR1 + (0.255795681) × VCP + (0.393370303) × MBTPS2 + (-0.292131004) × RHBDD2. Based on the median risk score, patients from the TCGA-LUAD cohort were stratified into a high-risk subgroup (n = 220) and a low-risk subgroup (n = 220) to facilitate further investigations. Subsequent K-M analysis and risk survival status plot revealed that the high-risk subgroup exhibited a worse prognosis, whereas the low-risk subgroup demonstrated prolonged survival (Fig. 4D and F). The prognostic models were assessed by calculating the area under the curve (AUC) for 1-year, 3-year, and 5-year survival, yielding values of 0.68, 0.69, and 0.70, respectively (Fig. 4E).

### Assessment and external validation for ERSRGs-signature

**Fig. 5:**
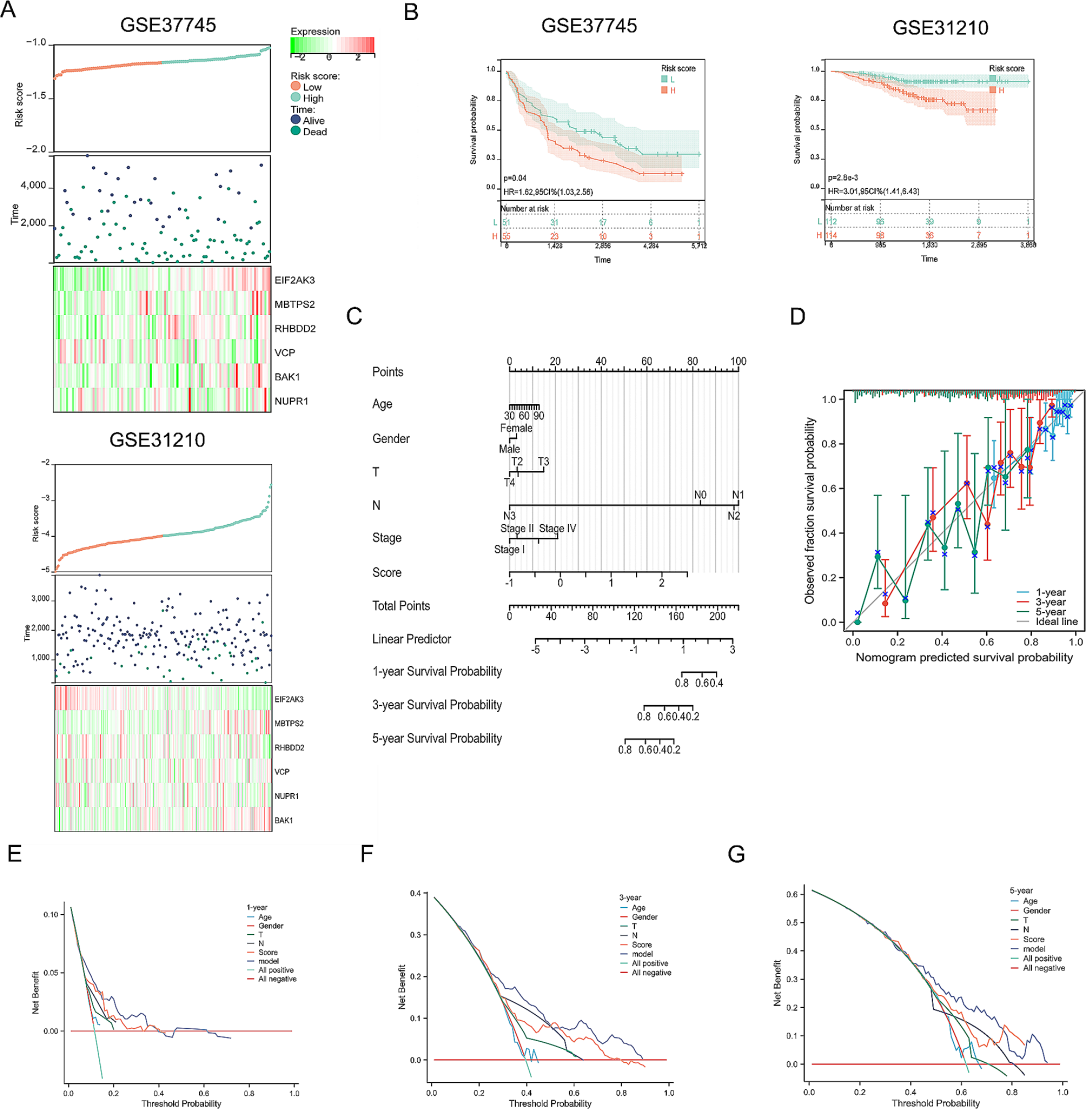
Assessment and external validation for ERSRGs-signature. (**A**) Riskscore plot of 6 ERSRGs-signature in external testing set, with riskscore and survival status in GSE37745 and GSE31210. (**B**) The Kaplan-Meier survival curves of high-risk and low-risk subgroups in external testing set. (**C**) Nomogram equipped with the riskscore and clinical parameters (age, gender, T, N and pathological stage) in TCGA. (**D**) The calibration curves displayed the accuracy of nomogram. (**E**-**G**) Decision curve analysis of nomogram (1-, 3-, 5- years)

The risk distribution curve, survival status, and expression heatmap of the external validation sets (GSE37745 and GSE30210) demonstrated that patients with low-risk scores exhibited significantly longer survival times compared to those with high-risk scores, thus validating the findings from the training set (Fig. 5A and B). To further consolidate the prognostic model, the clinical information and genetic characteristics from TCGA were integrated, and a comprehensive multi-factor Cox regression model was developed, resulting in the construction of a nomogram (Fig. 5C). Calibration plots were employed to assess the predictive accuracy of the nomogram, revealing excellent agreement between the predicted and observed OS rates at 1, 3, and 5 years (Fig. 5D). Moreover, the nomogram model was subjected to decision curve analysis (DCA) to evaluate its clinical utility and potential benefits (Fig. 5E-G). Collectively, the risk score, when combined with the ERSRGs-signature, pathological stage, and N-stage, emerged as an independent and robust prognostic indicator, providing enhanced prognostic value for patients with LUAD.

### Exploring immune infiltration patterns and single-cell analysis of ERSRGs-signature in LUAD

**Fig. 6:**
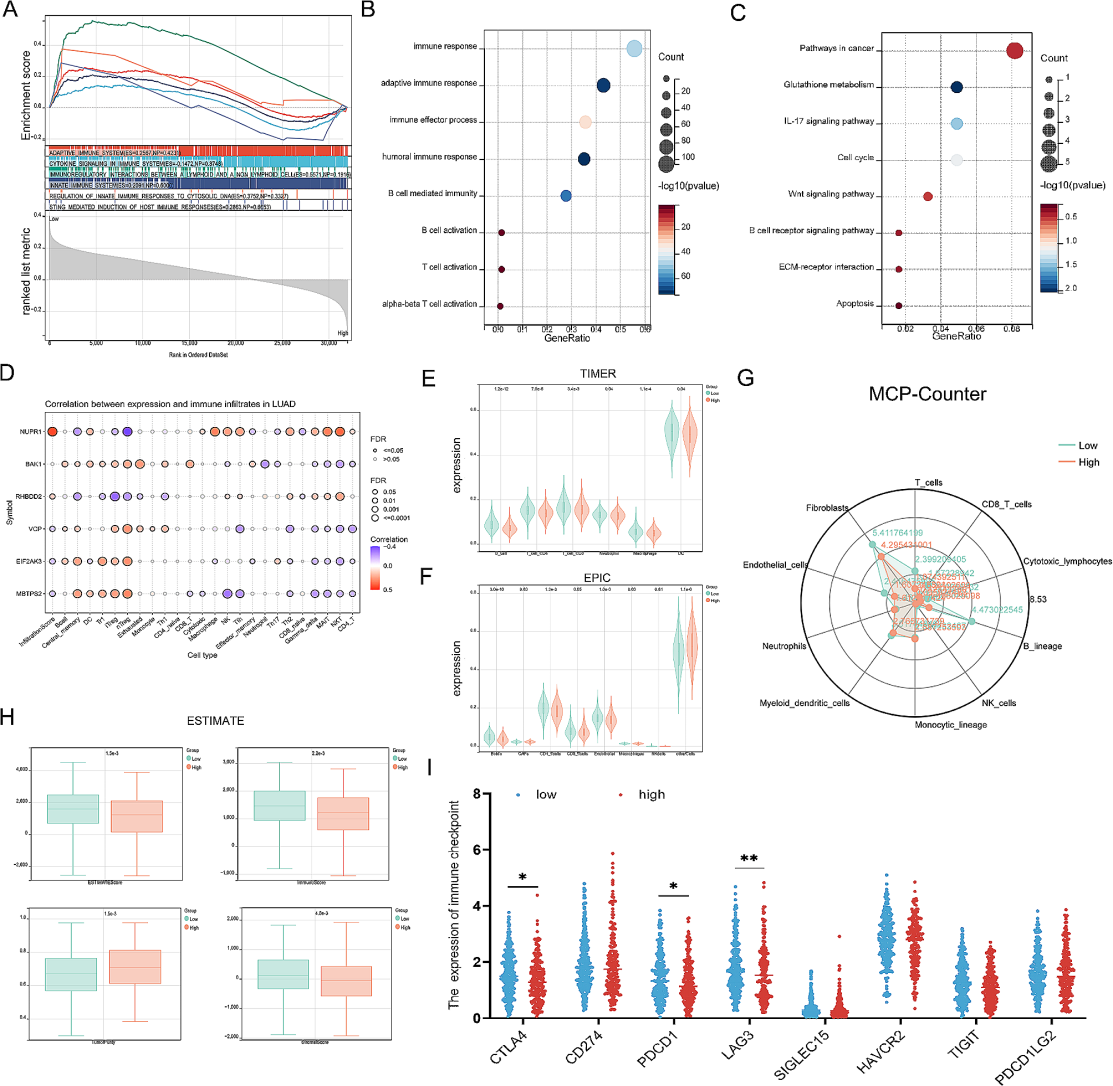
Immune infiltration analysis of ERSRGs-signature in LUAD. (**A**) The GSEA enrichment analysis between high riskscore subgroup and low riskscore subgroup. Analysis of GO (**B**) and KEGG (**C**) in differentially expressed genes. (**D**) The correlation between ERSRGs-expression and immune infiltrates. The TIMER (**E**), EPIC (**F**), MCP-Counter (**G**) and ESTIMATE (**H**) algorithm between high and low risk subgroups. (**I**) The expression of immune checkpoints was compared between the low vs. high riskscore subgroups. *P < 0.05, **P < 0.01

To unravel the potential functions and pathways associated with prognostic features, we conducted comprehensive enrichment analyses of Gene Set, GO, and KEGG pathways. The results revealed that the genes linked to prognostic features were predominantly enriched in pathways related to immunoinfiltration. Hence, we proceeded to explore the heterogeneity of immune microenvironments among ERSRGs-signature (Fig. 6A-C). Initially, we assessed the correlation between gene expression and immune infiltration in LUAD and observed significant variations in the expression of different genes across immune cells (Fig. 6D). Subsequently, we employed the TIMER and EPIC algorithms to investigate immune infiltration patterns between the low and high-risk subgroups. The low-risk subgroup exhibited significantly elevated expression of B cells, CD4 T cells, CD8 T cells, and macrophage cells compared to the high-risk group (Fig. 6E and F). To validate the stability and robustness of these findings, we utilized additional algorithms, namely MCP-counter and ESTIMATE, which yielded consistent results (Fig. 6G and H). Furthermore, we observed substantial differences in the expression of immune checkpoints between the two subgroups (Fig. 6I).

**Fig. 7:**
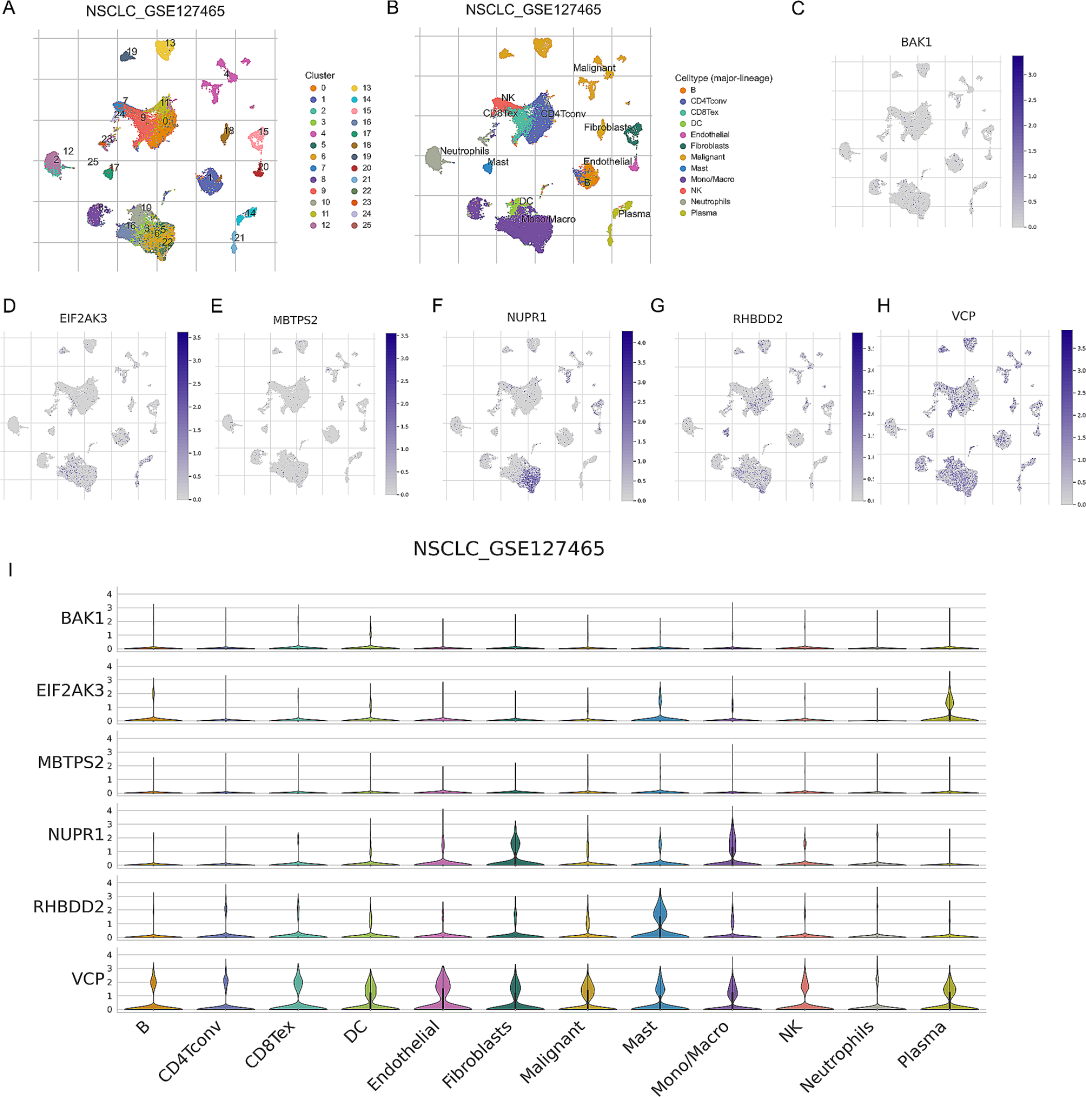
Single cell sequencing analysis of ERSRGs-signature. (**A**) tSNE clustering colored by groups. (**B**) The annotation of clusters based on marker analysis. mRNA distribution of BAK1 (**C**), EIF2AK3 (**D**), MBTPS2 (**E**), NUPR1 (**F**), RHBDD2 (**G**) and VCP (**H**) after tSNE dimensionality reduction. (**I**) Differential expression of ERSRGs in the different cell clusters

Subsequently, we conducted single-cell sequencing analysis of the ERSRGs-signature. Cluster analysis was performed, and Fig. 7A depicted the cluster display using t-distributed stochastic neighbor embedding (tSNE), where each color represented a distinct cell type identified within the clusters. Each cell was represented by a scatter plot, and the numbers in the figure corresponded to the cluster numbers. It was evident that there are 25 distinct cell populations. Figure 7B presented the annotation of clusters based on marker analysis, revealing significant differences in gene expression among different immune cells. After applying tSNE dimensionality reduction, the mRNA distribution of BAK1, EIF2AK3, MBTPS2, NUPR1, RHBDD2, and VCP was shown in Fig. 7C-H. Finally, we analyzed the differential expression of ERSRGs in the various immune cell clusters. Among them, BAK1 exhibited the lowest expression in immune cells, while VCP demonstrated the highest expression (Fig. 7I).

Overall, the riskscore demonstrated an inverse correlation with the level of immune infiltration, providing novel insights into the relationship between ERSRGs and the immune status of LUAD.

### Validation of the expression levels of ERSRGs in LUAD

**Fig. 8:**
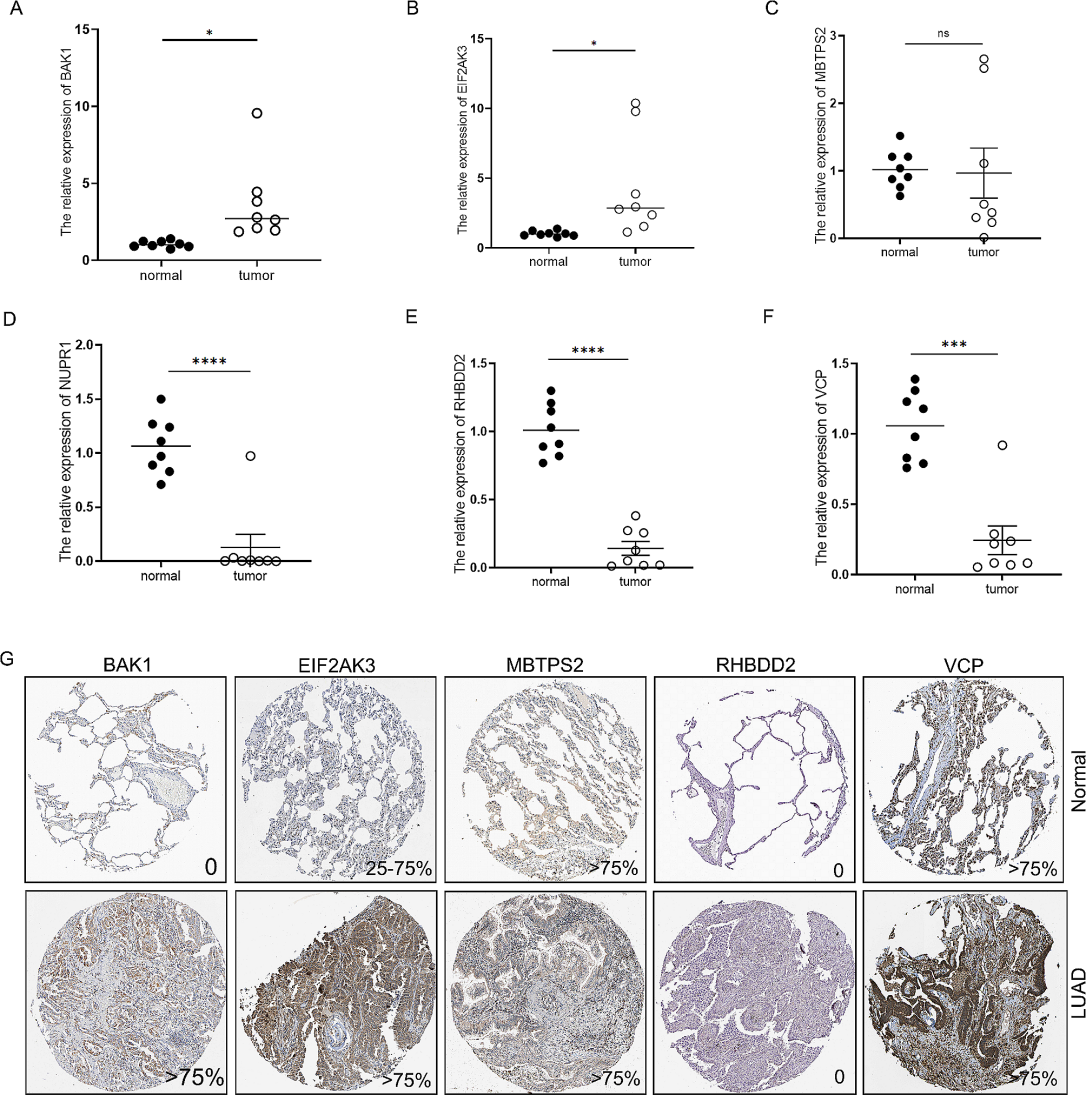
Validation of the expression levels of ERSRGs in LUAD. The mRNA expression of BAK1 (**A**), EIF2AK3 (**B**), MBTPS2 (**C**), NUPR1 (**D**), RHBDD2 (**E**) and VCP (**F**) in LUAD patients from Nantong tumor hospital. N = 8, ^*^P < 0.05, ^**^P < 0.01, ^***^P < 0.001, ^****^P < 0.0001. (**G**) The protein expression of BAK1, EIFAK3, MBTPS2, RHBDD2 and VCP in HPA

**Fig. 9:**
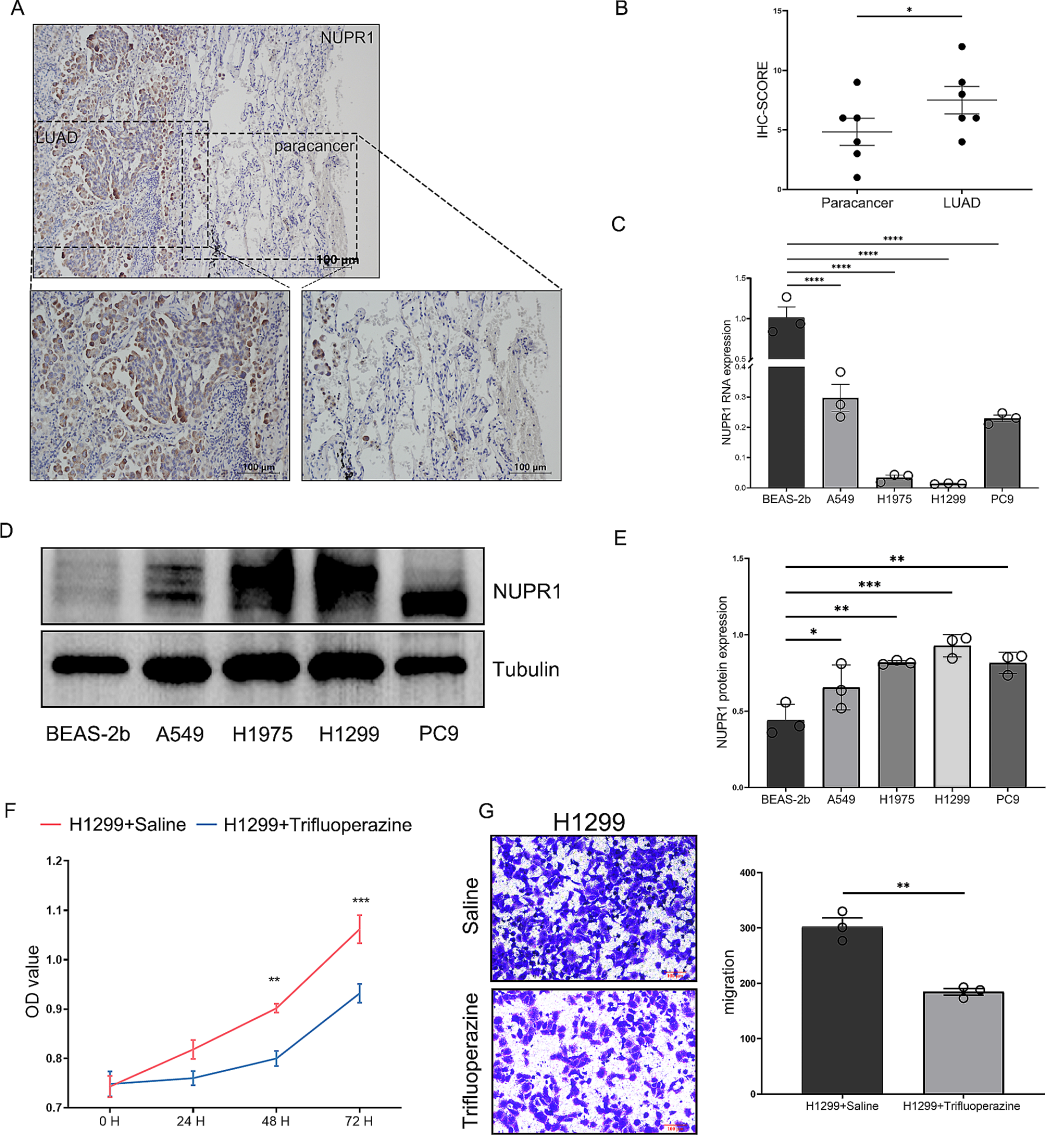
Expression analysis of NUPR1 at transcription and translation Levels. Representative images (**A**) and quantification (**B**) of NUPR1 in intratumoral and peritumoral fractions through immunohistochemistry staining (N = 6). MRNA (**C**) and protein expression (**D**&**E**) of NUPR1 in cell lines (N = 3). (**F**) Cell viability assessed through CCK8 assays between saline and trifluoperazine subgroups (N = 6). (**G**) Representative images and results of cell counting from the Transwell invasion assay (N = 3). ^*^P < 0.05, ^**^P < 0.01, ^***^P < 0.001, ^****^P < 0.0001

To further investigate the association between the prognostic ERSRGs-signature and LUAD, in vitro experiments were conducted using qPCR analysis on peritumoral and tumor tissues. The findings revealed a significant upregulation of BAK1 and EIF2AK3 expression in LUAD tissues, whereas NUPR1, RHBDD2, and VCP exhibited the opposite trend (Fig. 8A-G). Moreover, the Human Protein Atlas (HPA) database analysis showed higher expression levels of BAK1A and EIF2AK3 in LUAD tissues compared to normal tissues (Fig. 8G). However, NUPR1 data was unavailable in the HPA database. Therefore, to explore the protein expression of NUPR1 in LUAD patients, IHC analysis was performed at Nantong Cancer Hospital. Interestingly, the protein expression of NUPR1, as determined by IHC, exhibited an opposite pattern compared to the mRNA expression patterns (Fig. 9A).

### Validation of NUPR1 under experiment

In this study, we implemented a comprehensive validation of NUPR1 within authentic laboratory conditions. Initially, IHC analysis was conducted on pathological specimens obtained from 6 LUAD patients. The results revealed a conspicuous aggregation of NUPR1 within cancerous tissue compared to adjacent non-cancerous tissues (Fig. 9A and B). Subsequently, both RNA and protein expression levels of NUPR1 were scrutinized in normal lung epithelial cells and four distinct LUAD cell lines. Surprisingly, NUPR1 RNA exhibited its highest expression in normal cell lines (Fig. 9C), aligning with our bioinformatics analysis outcomes. In contrast, NUPR1 protein displayed heightened expression levels in LUAD cells (Fig. 9D and E, Fig. S2 and S3). We postulated that potential post-translational modifications may underlie this incongruity. To gain deeper insights into the functional role of NUPR1 in LUAD progression, we procured NUPR1 inhibitors and executed cell proliferation and transwell experiments. The results starkly indicated that upon NUPR1 inhibition, both cell proliferation and invasive capacity were markedly attenuated (Fig. 9F and G). This unequivocally underscores the contributory role of NUPR1 protein in the advancement of LUAD.

### Correlation between risk score and IC50 values for therapeutic agents

**Fig. 10:**
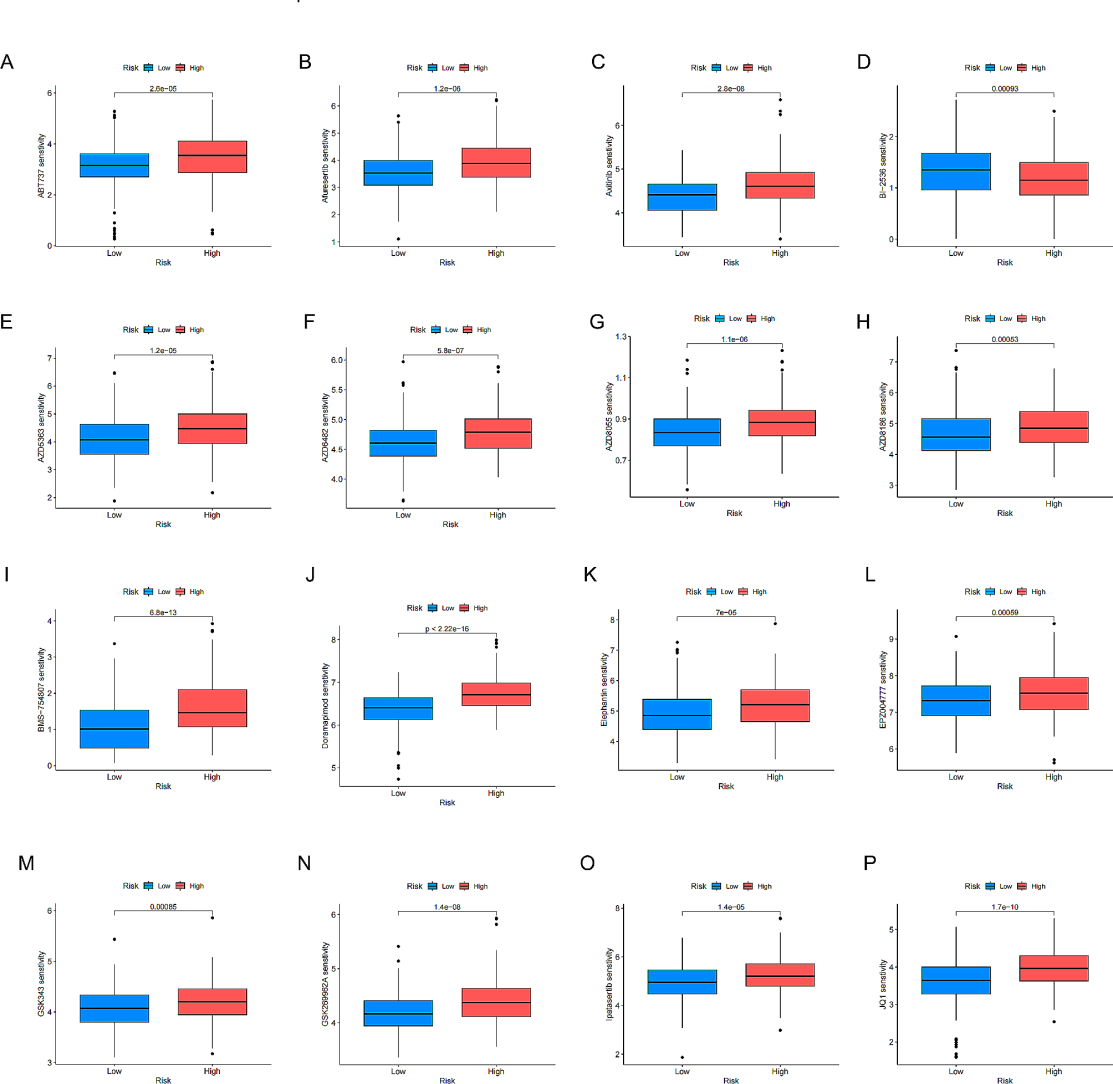
(**A**-**P**) Therapeutic drugs showed significant IC50 differences in high- and low-risk groups

The impact of risk scores on the IC50 values of a set of 30 distinct drug molecules was systematically assessed to discern their therapeutic efficacy. Except for BI-2536 and WIKI4, all other drugs exhibited higher resistance in the high-risk group (Fig. 10 and Fig. S1). This observation underscores the potential utility of our prognostic model in guiding the use of therapeutic agents.

## Discussion

LUAD represents the most prevalent subtype of lung cancer, a grave malignancy arising from the accumulation of various genetic mutations. These mutations lead to uncontrolled proliferation of lung cells and the subsequent formation of tumors. Upon recognition by the immune system, these transformed cancer cells elicit an immune response aimed at their elimination [21]. Nonetheless, immune escape not only expedites tumor progression but also impairs the efficacy of cancer immunotherapy [22, 23]. The ER pathway serves as a critical regulator of ER homeostasis. Disruption of ER function triggers a phenomenon referred to as “ER stress” [24]. In the context of tumorigenesis, the rapid proliferation rate of cancer cells necessitates heightened activity of ER protein folding, assembly, and transport, thereby inducing physiological stress within the ER [25]. The ER stress response is believed to confer cellular protection and is implicated in tumor growth and adaptation to challenging environments [26]. Sustained ER stress represents a novel characteristic of cancer, resulting from various metabolic and carcinogenic abnormalities that disrupt protein-folding homeostasis in aggressive immune cells. Constitutive activation of the ER stress response enables malignant cells to adapt to carcinogenesis and environmental stressors by coordinating multiple immune regulatory mechanisms and promoting malignant progression concurrently [27]. Nonetheless, the precise relationship between ER stress and the immune microenvironment remains inadequately investigated.

In our study, we initially screened 106 genes associated with ER stress to identify differential expression patterns between cancer and para-cancer samples. K-Medoids clustering was employed for this purpose. The differential genes in the two resulting clusters were primarily enriched in processes related to the adaptive immune system, humoral immune response, and regulation of humoral immune response. Notably, patients belonging to cluster 1 exhibited a significantly longer survival time compared to those in cluster 2. This discrepancy in prognosis suggests a potential correlation with immune response. Through a series of statistical analyses, including univariate regression, LASSO, and logistic stepwise regression, we identified 6 key ERSRGs. Subsequently, we constructed a novel prognostic risk spectrum based on the expression signature of these six genes (referred to as ERSRGs). This risk spectrum allowed us to classify patients with LUAD into distinct risk subgroups, based on their respective median risk scores. Importantly, a higher risk score was associated with worse prognosis for the patients.

The prognostic features of interest encompass 6 ERSRGs, specifically EIF2AK3, MBTPS2, RHBDD2, VCP, NUPR1, and BAK1. Among these, EIF2AK3, NUPR1, and RHBDD2 demonstrated protective characteristics, while MBTPS2, VCP, and BAK1 were strongly associated with poor prognosis. To assess their expression levels, qPCR analyses were conducted on cancer and para-cancer samples from 8 patients diagnosed with LUAD. The results revealed significant differential expression of EIF2AK3, RHBDD2, VCP, NUPR1, and BAK1, with NUPR1 and RHBDD2 exhibiting the most pronounced differences. EIF2AK3 has been identified as an immune-related prognostic gene in breast cancer, exerting a role in tumor cell apoptosis and facilitating sustained protective antitumor immunity [28]. MBTPS2, a membrane-embedded zinc metalloprotease, activates signaling proteins involved in transcriptional control of sterol and the ER stress response [29], thus promoting the progression of prostate cancer [30] and colorectal cancer [30]. The RHBDD2 (Rhomboid domain containing 2) gene is found to be overexpressed in advanced stages of colorectal cancer (CRC) and potentially modulates the UPR pathway, thereby favoring cell migration, adhesion, and proliferation [31]. VCP (valosin-containing protein) is crucial for maintaining mitochondrial function, and in prostate cancer cells, it employs self-aggregation to inhibit mitochondrial activity, thereby evading cell death during nutrient deprivation and promoting malignancy [32]. In a cohort study, Tao et al. demonstrated that NUPR1 serves as a protective factor in the survival prognosis of LUAD [33], while Li et al. suggested NUPR1 to be a potential risk gene [34]. NUPR1, a nuclear protein, plays a critical role in redox reactions [35], and macrophages have been implicated as the most relevant immune cells associated with NUPR1 expression in bladder cancer [36]. Furthermore, the mechanism through which BAK1 promotes cisplatin resistance in NSCLC is believed to involve the inhibition of cell apoptosis [37]. In summary, all 6 identified genes contribute to tumor development and progression by modulating pathways associated with tumor metabolism, with NUPR1 considered particularly significant.

Nuclear Protein 1 (NUPR1) is a small, highly basic transcriptional regulator involved in the regulation of diverse cellular processes, such as DNA repair, ER stress, and oxidative stress response. The cellular localization of NUPR1 appears to be associated with pathological conditions. Prominent cytoplasmic staining has been observed in large papillary tumors, tumors exhibiting lymph node metastasis, and NSCLC [38]. Our IHC analysis corroborated these findings. However, intriguingly, our real-world cohort study revealed that, in contrast to mRNA expression, NUPR1 accumulates in cancerous tissues, contributing to the malignant progression of cancer, which necessitates further investigation. Garcia Montero et al. reported that under various stress conditions, NUPR1 mRNA expression was rapidly, strongly, and transiently stimulated [39]. Cancer cells endure and adapt to various types of stressful environments over prolonged periods [40], leading us to speculate that NUPR1 mRNA may be consumed more in cancerous tissues compared to adjacent tissues. Additionally, interestingly, the protein expression of NUPR1 has been shown to positively correlate with cell density [41]. Considering that cancer arises from unregulated and excessive cell division and proliferation, resulting in higher cell density [42], we hypothesize that NUPR1 expression is relatively elevated in cancer cells characterized by higher cell density compared to adjacent cells with relatively fewer cells.

To verify the broad applicability of the risk assessment element group, we conducted validation using external datasets GSE31210 and GSE37745. The signature exhibited robust predictive performance not only in the internal dataset but also in the validation sets. Evidence from ROC curves and K-M analysis demonstrated the remarkable predictive effect of the ERSRGs on the prognosis of LUAD patients. Importantly, even after stratifying clinical features, this signature remained significantly prognostic in LUAD patients. Therefore, we propose that ER stress-related features possess excellent predictive performance for OS and could serve as independent prognostic indicators for LUAD. To facilitate clinical application, we constructed a nomogram model and verified its accuracy using calibration diagrams.

Previous research has highlighted the role of ER stress in promoting immune escape and facilitating metastasis [43, 44]. Subsequent GSEA, GO, and KEGG analyses of the two subgroups revealed enrichment in immune-related pathways. Notably, tumor purity has been identified as negatively correlated with immune response, suggesting its potential as an indicator of the immune response level in the tumor microenvironment [45]. To explore this further, we employed four different immune scoring algorithms, and all results consistently indicated that individuals classified as low-risk exhibited higher expression levels of B cells, CD4^+^ T cells, CD8^+^ T cells, neutrophils, macrophages, and endothelial cells. The density of CD8^+^ T cells and mature dendritic cells has been closely associated with the survival rate of lung cancers, with higher CD8^+^ T cell density correlating with better 5-year survival rates [46], consistent with our findings. Additionally, we observed decreased expression of immune checkpoint genes in the high-risk group, which may be attributed to immune cell dysregulation. Therefore, our new prognostic model holds potential to not only assess the survival prognosis of LUAD but also shed light on the immune microenvironment.

Several limitations should be acknowledged in this study. Firstly, the model primarily relies on data from the TCGA database and the Nantong cohort, thus its generalizability to other datasets may be limited. Therefore, a prospective multicenter cohort study is necessary to validate the findings and ensure their applicability to diverse populations. Secondly, in order to comprehensively elucidate the underlying reasons for the discordance between NUPR1 mRNA and protein expression levels, further evidence from additional experiments and investigations is required.

Overall, this study presents a prognostic model based on six genes associated with ER stress. The model exhibits utility in predicting the survival outcomes of patients with LUAD and offers insights into tumor immune infiltration to some extent. Furthermore, the identification of key genes provides novel insights into the molecular mechanisms underlying LUAD.

### Electronic supplementary material

Below is the link to the electronic supplementary material.

**Supplementary Material 1: Fig. S1** (A-N) Therapeutic drugs showed significant IC50 differences in high- and low-risk groups. **Fig. S2** Original, unprocessed versions for WB: NUPR1. **Fig. S3** Original, unprocessed versions for WB: Tubulin

## Data Availability

The datasets generated during and/or analyzed during the current study are available in the TCGA (http://www.genome.ucsc.edu/) and GEO (https://www.ncbi.nlm.nih.gov/geo/) Databases, with specific accession numbers GSE31210 and GSE37745. The original contributions presented in the study are included in the article/Additional files, and further inquiries can be directed to the corresponding authors.
