# Supplementary material for: Integrated multi-omic analysis and experiment reveals the role of endoplasmic reticulum stress in lung adenocarcinoma
Source: BMC Med Genomics. 2024 Jan 2;17:12. doi: 10.1186/s12920-023-01785-4 (PMC10763289; doi:10.1186/s12920-023-01785-4)
Supplement: Supplementary file 1 — Supplementary Material 1: Fig. S1 (A-N) Therapeutic drugs showed significant IC50 differences in high- and low-risk groups. Fig. S2 Original, unprocessed versions for WB: NUPR1. Fig. S3 Original, unprocessed versions for WB: Tubulin [file 12920_2023_1785_MOESM1_ESM.docx]

**Supplement information**


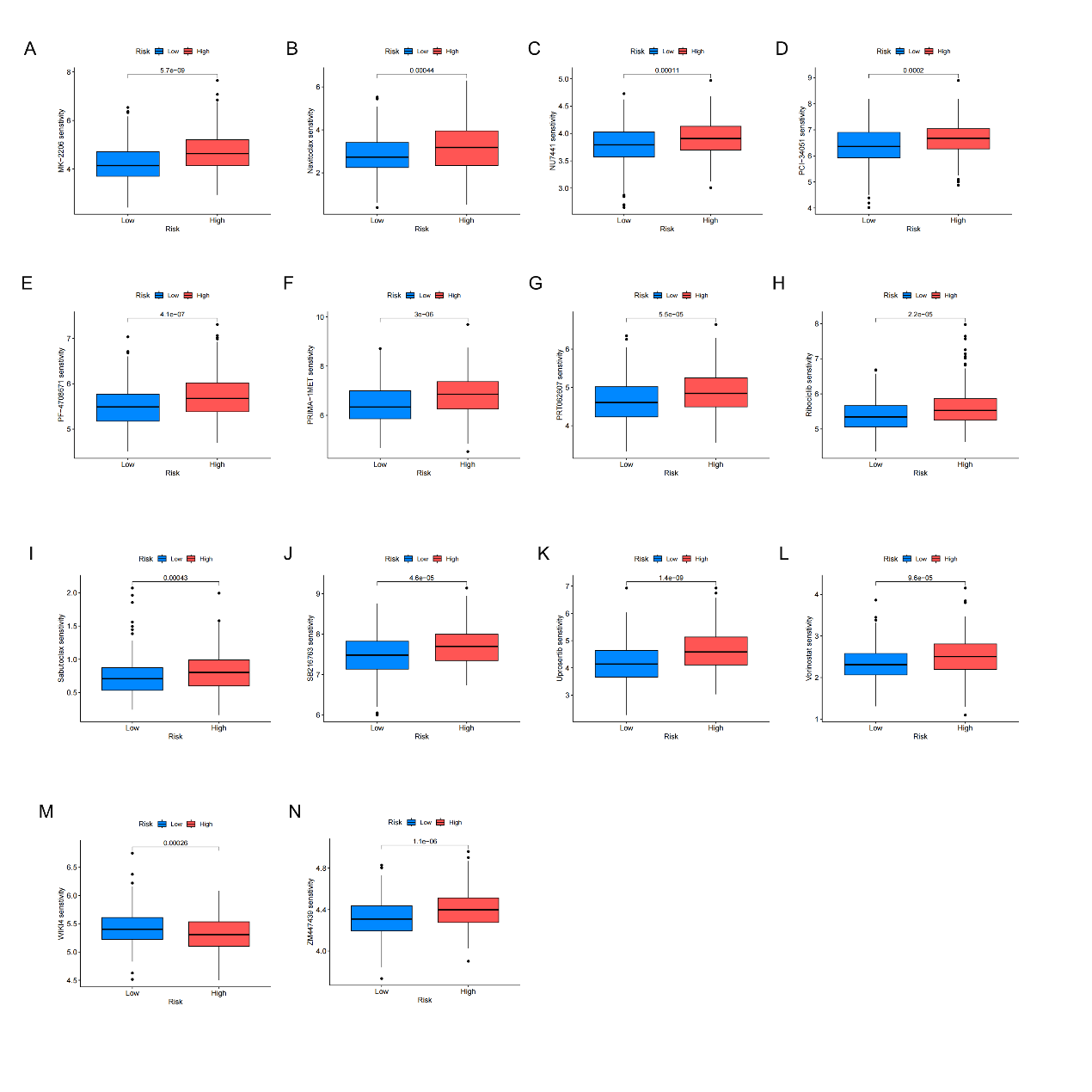


**Fig. S1** (A-N) Therapeutic drugs showed significant IC50 differences in high- and low-risk groups.


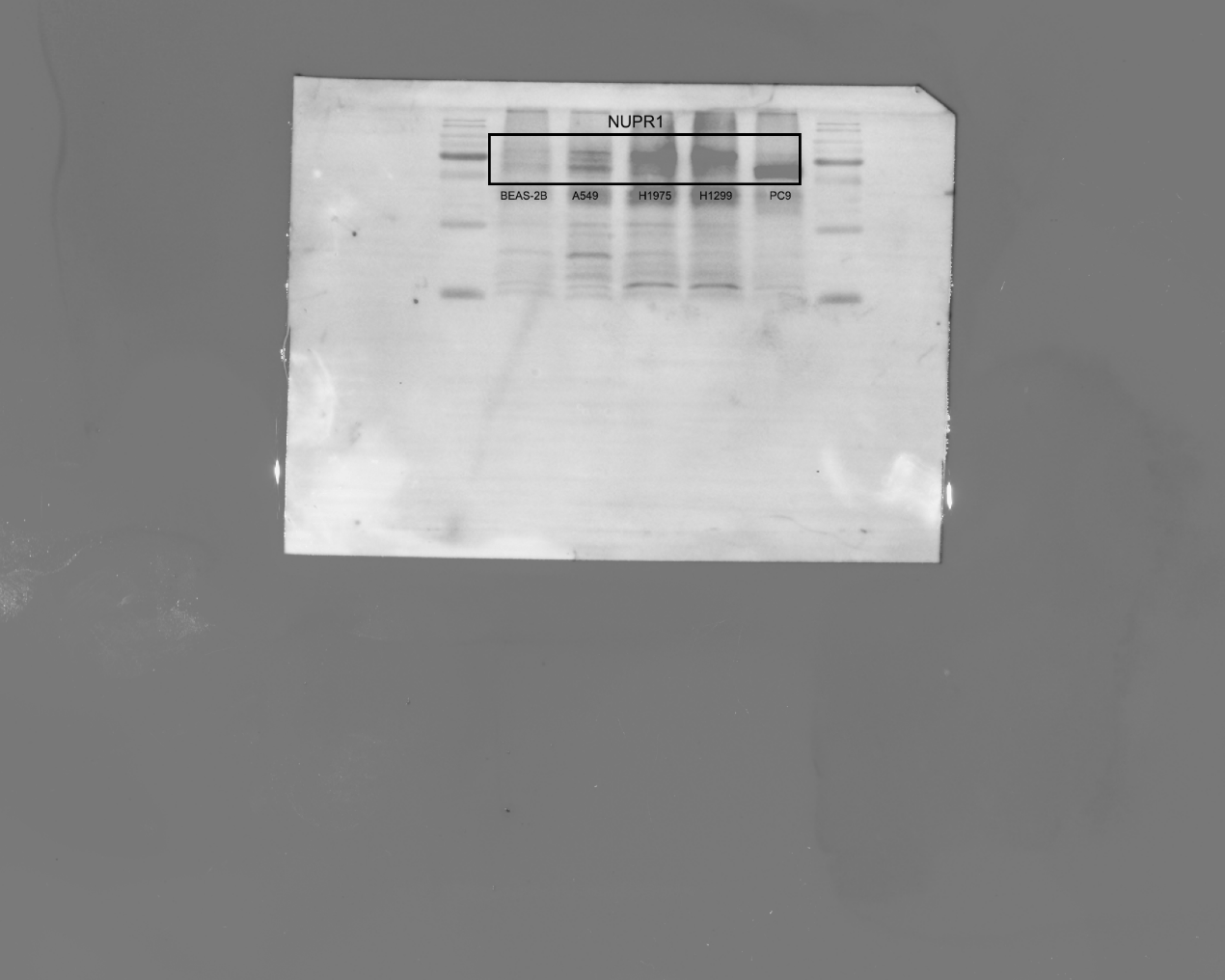


**Fig. S2** Original, unprocessed versions for WB: NUPR1.


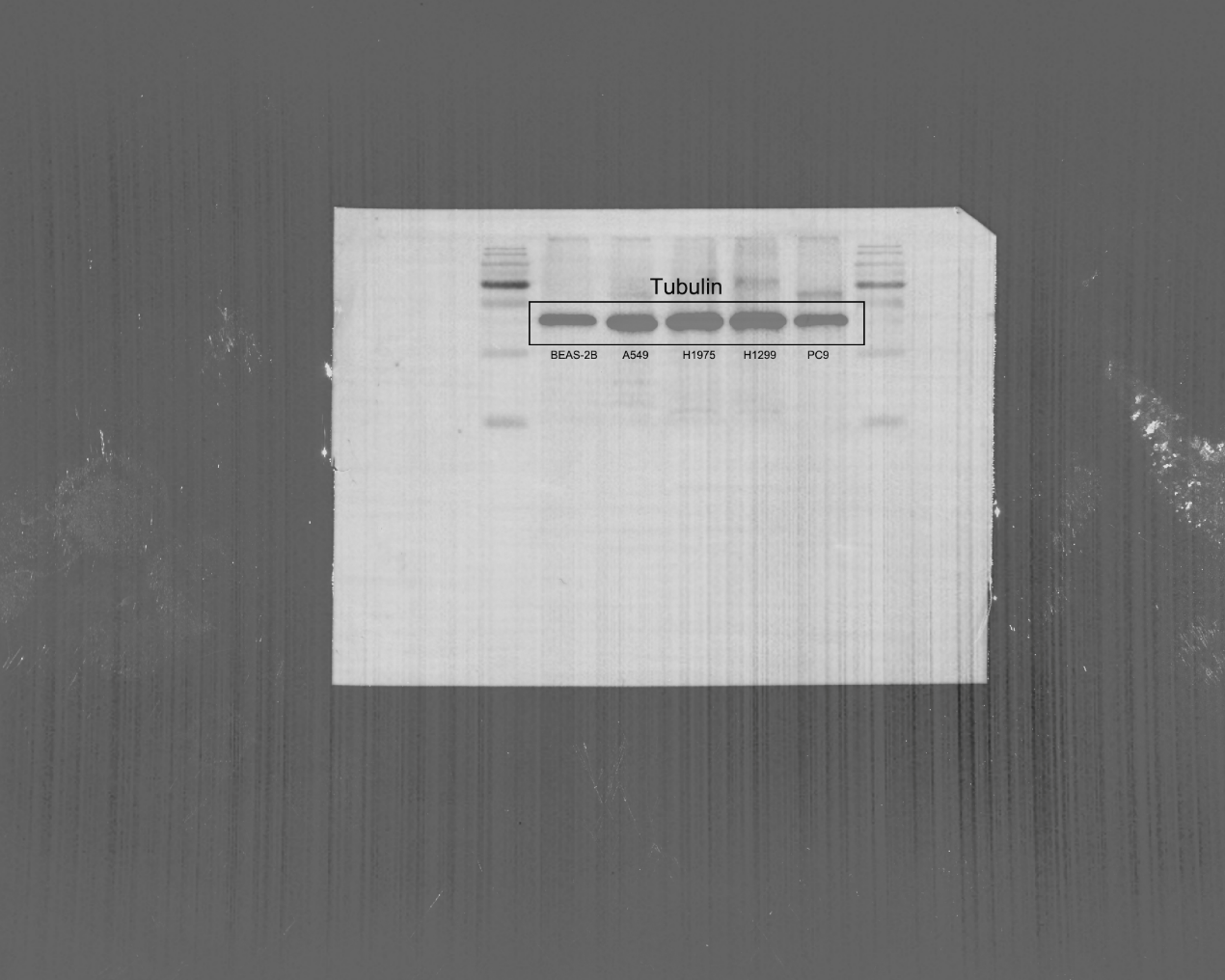


**Fig. S3** Original, unprocessed versions for WB: Tubulin
